# Supplementary material for: Structural Characteristics of High-Mobility Group Proteins HMGB1 and HMGB2 and Their Interaction with DNA
Source: Int J Mol Sci. 2023 Feb 10;24(4):3577. doi: 10.3390/ijms24043577 (PMC9962726; doi:10.3390/ijms24043577)
Supplement: Supplementary file 1 [file ijms-24-03577-s001.zip › ijms-2148811-supplementary.pdf]

Figure S1.

Electrophoresis of HMGB1 and HMGB2 isolated from calf thymus.

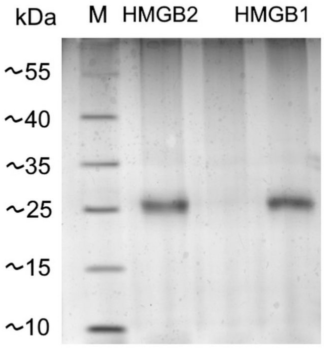

Figure S2.

(a) Schematic representation of a protocol for the extraction of HMGB1 and HMGB2 from mammalian tissue.

(b) Denaturing electrophoresis of HMGB1/2-rich extracts from calf thymus.

(c) Identification of HMGB1 and HMGB2 proteins from calf thymus by Western Blot.

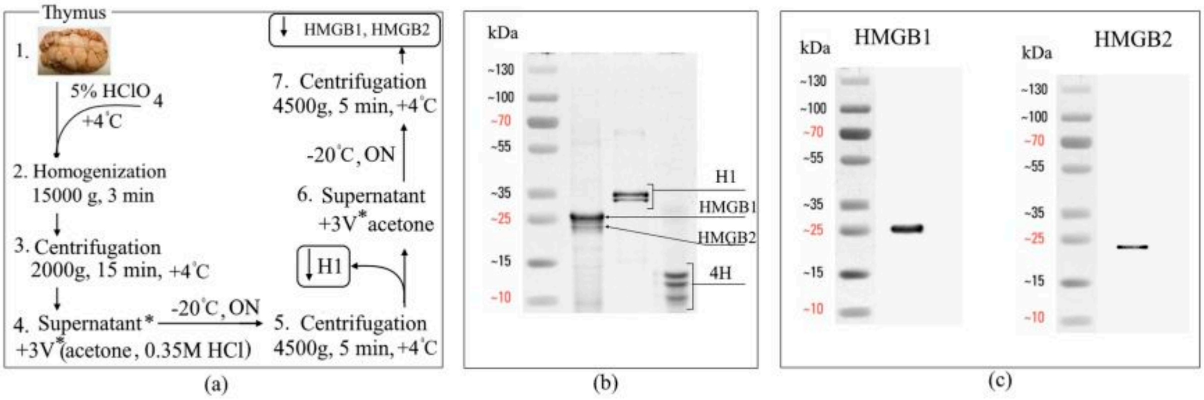

Figure S3. Mass spectra of HMGB1 and HMGB2 from calf thymus. Mass spectra were recorded using a Fourier Transform Ion Cyclotron Resonance Mass Spectrometer (MALDI-FT-ICR-MS, Varian) in the mass range 500–3000 m/z. The obtained mass spectra were processed using the ProteinProspector and Mascot software packages.

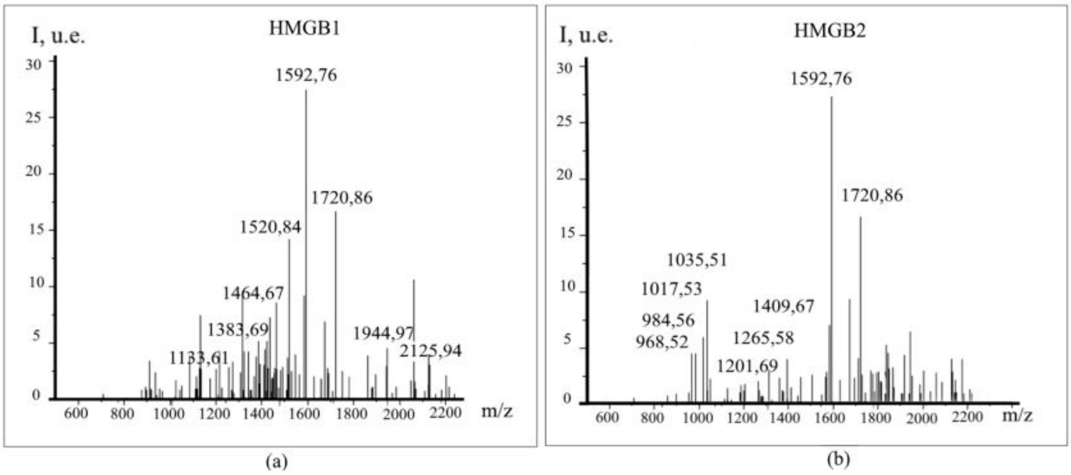

Figure S4. Map of the PTMs regions for the HMGB1 and HMGB2 proteins and comparison with the literature data [55,58].

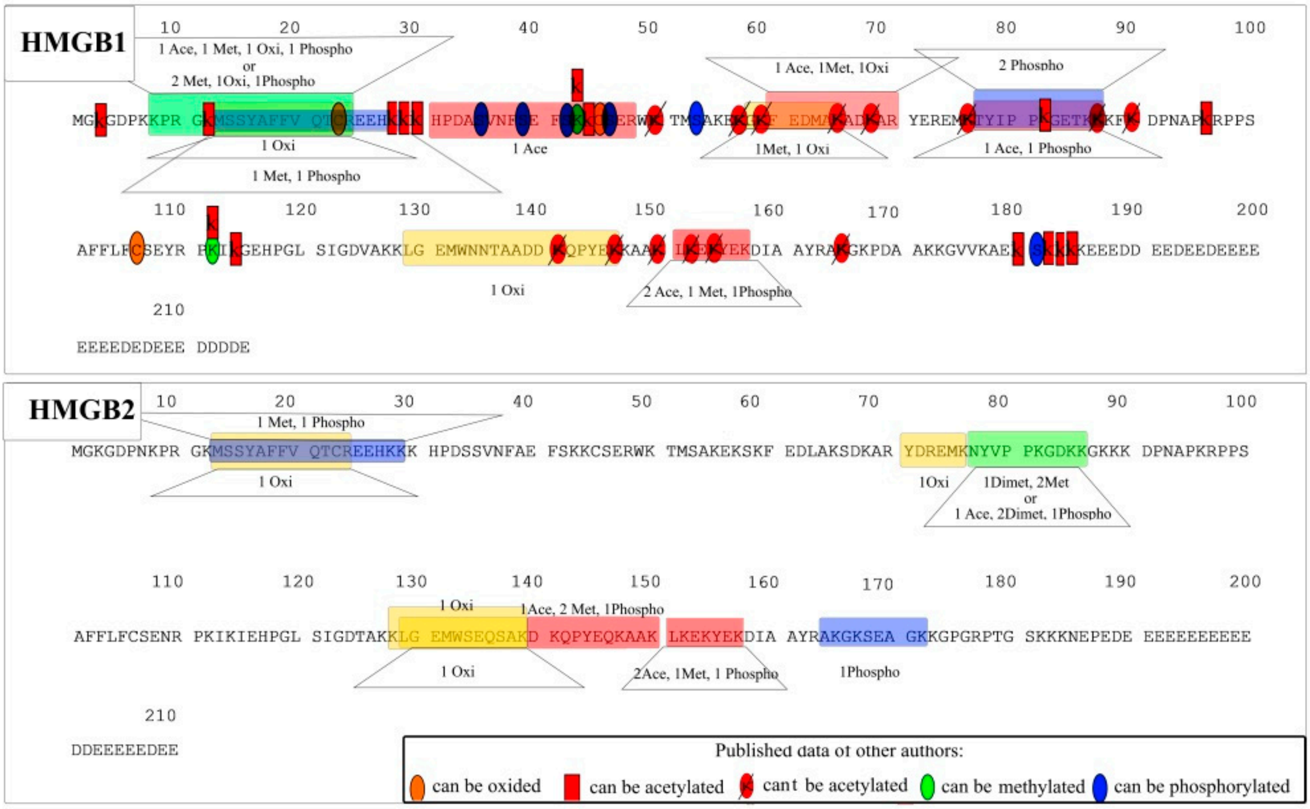

Table S1. The results of MALDI mass spectrometry analysis of HMGB1 and HMGB2 from calf thymus.

protein	m/z	I	m/z teor	ppm	modification	peptide	sequence
HMGB2	708,366947	0,3540	708,3675	0,78		158-163	(K)DIAAYR(A)
HMGB1	708,366947	0,3540	708,3675	0,78		158-163	(K)DIAAYR(A)
HMGB2	857,382193	0,5930	857,3822	0,01	1Oxidation	71-76	(R)YDREMK(N)
HMGB2	897,515494	0,7370	897,5152	0,33		89-96	(K)KKDPNAPK(R)
HMGB1	916,488975	0,8600	916,4887	0,30		89-96	(K)FKDPNAPK(R)
HMGB1	941,438613	0,3260	941,4397	1,15	1Oxidation	58-65	(K)GKFEDMAK(A)
HMGB2	955,455764	0,8400	955,4608	5,27	1Phospho	164-172	(R)AKGKSEAGK(K)
HMGB1	955,455764	0,8400	955,4553	0,49	1Methyl 1Oxidation	58-65	(K)GKFEDMAK(A)
HMGB2	968,529299	4,4120	968,5272	2,17		4-12	(K)GDPNKPRGK(M)
HMGB2	984,560357	4,3090	984,5585	1,89		173-182	(K)KGPGRPTGSK(K)
HMGB2	1115,537124	0,3640	1115,5384	1,14	2Acetyl 1Methyl 1Phospho	151-157	(K)LKEKYEK(D)
HMGB1	1115,537124	0,3640	1115,5384	1,14	2Acetyl 1Methyl 1Phospho	151-157	(K)LKEKYEK(D)
HMGB2	1017,5379	5,8660	1017,5364	1,47		77-85	(K)NYVPPKGDK(K)
HMGB2	1035,510367	9,1470	1035,5106	0,23		140-147	(K)DKQPYEQK(A)
HMGB2	1128,571016	0,2590	1128,5684	2,32		155-163	(K)YEKDIAAYR(A)
HMGB1	1128,571016	0,2590	1128,5684	2,32		155-163	(K)YEKDIAAYR(A)
HMGB1	1133,61991	2,6130	1133,6201	0,17		77-86	(K)TYIPPKGETK(K)
HMGB2	1145,632766	0,2400	1145,6313	1,28		77-86	(K)NYVPPKGDKK(G)
HMGB2	1201,692253	1,0570	1201,6939	1,37	1Dimethyl 2Methyl	77-86	(K)NYVPPKGDKK(G)
HMGB2	1265,584401	1,8770	1265,5831	1,03		129-139	(K)LGEMWSEQSAK(D)
HMGB2	1279,662849	0,5250	1279,6641	0,98		60-70	(K)FEDLAKSDKAR(Y)
HMGB1	1279,662849	0,5250	1279,6641	0,98		115-127	(K)GEHPGLSIGDVAK(K)
HMGB2	1281,577019	0,5560	1281,5780	0,77	1Oxidation	129-139	(K)LGEMWSEQSAK(D)
HMGB2	1323,672416	0,2720	1323,6708	1,22	1Acetyl 2Dimethyl 1Phospho	77-86	(K)NYVPPKGDKK(G)
HMGB1	1353,647141	0,2720	1353,6467	0,33	1Acetyl 1Methyl 1Oxidation	60-70	(K)FEDMAKADKAR(Y)
HMGB1	1383,690879	5,1060	1383,6920	0,81	1Acetyl 1Phospho	77-87	(K)TYIPPKGETKK(K)
HMGB2	1393,678039	0,2770	1393,6780	0,03		128-139	(K)KLGEMWSEQSAK(D)
HMGB2	1409,672726	1,3880	1409,6729	0,12	1Oxidation	128-139	(K)KLGEMWSEQSAK(D)
HMGB1	1421,654057	0,6890	1421,6477	4,47	2Phospho	77-87	(K)TYIPPKGETKK(K)
HMGB2	1439,644988	0,6530	1439,6446	0,27		13-24	(K)MSSYAFFVQTCR(E)
HMGB1	1439,644988	0,6530	1439,6446	0,27		13-24	(K)MSSYAFFVQTCR(E)
HMGB2	1455,640633	2,2720	1455,6395	0,78	1Oxidation	13-24	(K)MSSYAFFVQTCR(E)
HMGB1	1455,640633	2,2720	1455,6395	0,78	1Oxidation	13-24	(K)MSSYAFFVQTCR(E)
HMGB2	1455,690652	0,8010	1455,6879	1,89	1Acetyl 2Methyl 1Phospho	140-150	(K)DKQPYEQKAAK(L)
HMGB1	1464,67626	8,5510	1464,6754	0,59		31-43	(K)HPDASVNFSEFSK(K)
HMGB1	1520,842667	14,1540	1520,8431	0,28		113-127	(K)IKGEHPGLSIGDVAK(K)
HMGB2	1592,769672	27,4630	1592,7703	0,39		30-43	(K)KHPDSSVNFAEFSK(K)
HMGB1	1592,769672	27,4630	1592,7703	0,39		31-44	(K)HPDASVNFSEFSKK(C)
HMGB1	1592,769672	27,4630	1592,7703	0,39		30-43	(K)KHPDASVNFSEFSK(K)
HMGB1	1720,865299	16,6560	1720,8653	0,00		29-43	(K)KKHPDASVNFSEFSK(K)
HMGB1	1720,865299	16,6560	1720,8653	0,00		30-44	(K)KHPDASVNFSEFSKK(C)
HMGB2	1720,865299	16,6560	1720,8653	0,00		29-43	(K)KKHPDSSVNFAEFSK(K)
HMGB2	1720,865299	16,6560	1720,8653	0,00		30-44	(K)KHPDSSVNFAEFSKK(C)
HMGB2	1741,743191	0,9160	1741,7420	0,68	1Dimethyl 1Methyl 2Phospho	71-82	(R)YDREMKNYVPPK(G)
HMGB1	1944,976296	4,4380	1944,9789	1,34		97-112	(K)RPPSAFFLFCSEYRPK(I)
HMGB1	2109,963844	0,6290	2109,9658	0,93	1Acetyl	31-48	(K)HPDASVNFSEFSKKCSER(W)
HMGB1	2125,947941	3,9420	2125,9495	0,73	1Oxidation	129-146	(K)LGEMWNNTAADDKQPYEK(K)

HMGB1	2130,002003	2,9790	2130,0024	0,19	2Methyl 1Oxidation 1Phospho	8-24	(K)KPRGKMSSYAFFVQTCR(E)
HMGB1	2157,996842	0,3960	2157,9964	0,20	1Acetyl 1Methyl 1Oxidation 1Phospho	8-24	(K)KPRGKMSSYAFFVQTCR(E)
HMGB2	2184,962069	0,7640	2184,9606	0,67	1Methyl 1Phospho	13-29	(K)MSSYAFFVQTCREEHKK(K)
HMGB1	2184,962069	0,7640	2184,9606	0,67	1Methyl 1Phospho	13-29	(K)MSSYAFFVQTCREEHKK(K)
HMGB1	2238,054522	0,4500	2238,0496	2,20		128-146	(K)KLGEMWNNTAADDKQPYEK(K)
HMGB1	2238,054522	0,4500	2238,0496	2,20		129-147	(K)LGEMWNNTAADDKQPYEKK(A)
